# Chemical efficacy of several NaOCl concentrations on biofilms of different architecture: new insights on NaOCl working mechanisms

**DOI:** 10.1111/iej.13198

**Published:** 2019-08-31

**Authors:** X. Petridis, F. H. Busanello, M. V. R. So, R. J. B. Dijkstra, P. K. Sharma, L. W. M. van der Sluis

**Affiliations:** ^1^ Department of Conservative Dentistry, Center for Dentistry and Oral Hygiene University Medical Center Groningen, University of Groningen Groningen The Netherlands; ^2^ Conservative Dentistry Department, School of Dentistry Federal University of Rio Grande do Sul Porto Alegre Brazil; ^3^ Department of Biomedical Engineering University Medical Center Groningen, University of Groningen Groningen The Netherlands

**Keywords:** biofilm, concentration, NaOCl, optical coherence tomography, removal, structure

## Abstract

**Aim:**

To investigate the anti‐biofilm efficacy and working mechanism of several NaOCl concentrations on dual‐species biofilms of different architecture as well as the changes induced on the architecture of the remaining biofilms.

**Methodology:**

*Streptococcus oralis* J22 and *Actinomyces naeslundii* T14V‐J1 were co‐cultured under different growth conditions on saliva‐coated hydroxyapatite discs. A constant‐depth film fermenter (CDFF) was used to grow steady‐state, four‐day mature biofilms (dense architecture). Biofilms were grown under static conditions for 4 days within a confined space (less dense architecture). Twenty microlitres of buffer, 2‐, 5‐, and 10% NaOCl were applied statically on the biofilms for 60 s. Biofilm disruption and dissolution, as well as bubble formation, were evaluated with optical coherence tomography (OCT). The viscoelastic profile of the biofilms post‐treatment was assessed with low load compression testing (LLCT). The bacteria/extracellular polysaccharide (EPS) content of the biofilms was examined through confocal laser scanning microscopy (CLSM). OCT, LLCT and CLSM data were analysed through one‐way analysis of variance (ANOVA) and Tukey’s HSD *post‐hoc* test. Linear regression analysis was performed to test the correlation between bubble formation and NaOCl concentration. The level of significance was set at *a* < 0.05.

**Results:**

The experimental hypothesis according to which enhanced biofilm disruption, dissolution and bubble formation were anticipated with increasing NaOCl concentration was generally confirmed in both biofilm types. Distinct differences between the two biofilm types were noted with regard to NaOCl anti‐biofilm efficiency as well as the effect that the several NaOCl concentrations had on the viscoelasticity profile and the bacteria/EPS content. Along with the bubble generation patterns observed, these led to the formulation of a concentration and biofilm structure‐dependent theory of biofilm removal.

**Conclusions:**

Biofilm architecture seems to be an additional determining factor of the penetration capacity of NaOCl, and consequently of its anti‐biofilm efficiency.

## Introduction

Sodium hypochlorite (NaOCl) is the main irrigant of choice during root canal treatment, with concentrations employed ranging between 0.5% and 6% (Slaus & Bottenberg 2002, Zehnder 2006, Dutner *et al.* 2012, Savani *et al.* 2014, Willershausen *et al.* 2015). Even though higher concentrations have been associated with improved treatment outcome, the level of evidence is weak (Fedorowicz *et al.* 2012). With randomized controlled clinical trials still in progress, the current lack of a definitive association between NaOCl concentration and treatment outcome calls for exploration of surrogate indicators that could provide criteria for selecting the desired concentration. Given that apical periodontitis is a biofilm‐induced disease (Ricucci & Siqueira 2010), the anti‐biofilm capacity of several NaOCl concentrations could serve that purpose.

Studies employing several biofilm models have shown a tendency towards increased biofilm removal with increasing NaOCl concentration (Arias‐Moliz *et al.* 2009, Retamozo *et al.* 2010, Jiang *et al.* 2011, Del Carpio‐Perochena *et al.* 2011). However, contradictory results have been reported when lower NaOCl concentrations are applied. One per cent NaOCl has been shown to partially disrupt and decrease the viability of a biofilm (Chávez de Paz *et al.* 2010, Del Carpio‐Perochena *et al.* 2011), whereas less or no effect at all has also been reported (Retamozo *et al.* 2010, Ordinola‐Zapata *et al.* 2012).

The lack of standardization in biofilm models (Swimberghe *et al.* 2018), limitations associated with post‐treatment biofilm analysis and the various ways that NaOCl is delivered in laboratory studies, could account for the discrepancies observed. For instance, biofilm architecture has been reported to play an important role in the removal of NaOCl‐induced biofilm removals (Busanello *et al.* 2019). Accordingly, this factor should be taken into account and standardized when biofilm models are designed. Developing dual‐species biofilms with various architectures is feasible by letting biofilms grow for four days under well‐defined growth conditions (Busanello *et al.* 2019).

In addition, from a biofilm analysis point of view, it has been demonstrated that structural alterations can be visualized and measured by means of optical coherence tomography (OCT); this is achieved by measuring the shifting that occurs at the greyscale level in pre‐ and post‐treatment greyscale images of biofilms acquired with the OCT (Haisch & Niessner 2007, Busanello *et al.* 2019, Petridis *et al.* 2019). Moreover, OCT allows for real‐time visualization and recording of the biofilm response to biocides (Rasmussen *et al.* 2016, Busanello *et al.* 2019), thus providing information on the working action of chemical solutions (Busanello *et al.* 2019). For NaOCl in particular, this is an important analytical feature since its anti‐biofilm working mechanism is largely unexplored.

As far as the NaOCl delivery is concerned, in the majority of relevant studies biofilms interact with an excess of NaOCl solution that surrounds the samples. Within the root canal system though, the area of contact between the biocide and the biofilm is rather limited. Therefore, a reduced contact surface area and limited NaOCl accessibility only to the top layer of the biofilm seem more realistic from a clinical standpoint.

Biofilms can survive NaOCl treatment (Stewart *et al.* 2001) resulting in post‐treatment biofilm persistence (Nair *et al.* 2005, Ricucci & Siqueira 2010). Depending on the environmental conditions, the remaining biofilm can re‐grow (Chávez de Paz *et al.* 2008, Shen *et al.* 2010, Ohsumi *et al.* 2015, Shen *et al.* 2016) and thereby perpetuate periapical disease (Siqueira & Rôças 2008). Due to the potential impact of recalcitrant biofilm on treatment outcome, investigating aspects of its structure could aid in the development of effective removal regimes (Peterson *et al.* 2015). One of the main structural features of biofilms is viscoelasticity. Biofilm viscoelasticity has been shown to correlate to biocide penetration and bacterial killing in oral biofilms (He *et al.* 2013, Rozenbaum *et al.* 2019); this has led to its acknowledgement as a virulence factor (Peterson *et al.* 2015,). Furthermore, low load compression testing (LLCT)‐based viscoelastic analysis of dual‐species biofilms with different architecture has provided interesting data on the viscoelastic profile of remaining biofilms, especially after NaOCl treatment (Busanello *et al.* 2019, Petridis *et al.* 2019). Lastly, confocal laser scanning microscopy (CLSM)‐aided evaluation of stained biofilm components (e.g. bacteria, extracellular polysaccharides‐EPS‐) on remaining biofilms post‐treatment contributes to the evaluation of the biofilm architecture as well (Busanello *et al.* 2019, Petridis *et al.* 2019).

This study aimed at evaluating the anti‐biofilm efficacy of several NaOCl concentrations on dual‐species biofilms of different architecture. The primary objective was to assess by means of OCT the biofilm disruption and dissolution mediated by the static application of 2‐, 5‐, and 10% NaOCl on four‐day grown dual‐species biofilms comprised of clinical isolates of *Streptococcus oralis* and *Actinomyces naeslundii*, and of different structural architecture. A secondary objective was to assess the effect of the same NaOCl concentrations on the architecture biofilms post‐treatment. This was achieved through evaluating their viscoelastic properties by means of LLCT and quantifying the changes in the proportion of stained biofilm components (live/dead bacteria and EPS) by means of confocal laser scanning microscopy (CLSM). The tertiary objective was to image real‐time by means of OCT the anti‐biofilm working action of the same concentrations of NaOCl during subtle flow.

## Materials and methods

**Figure 1:**
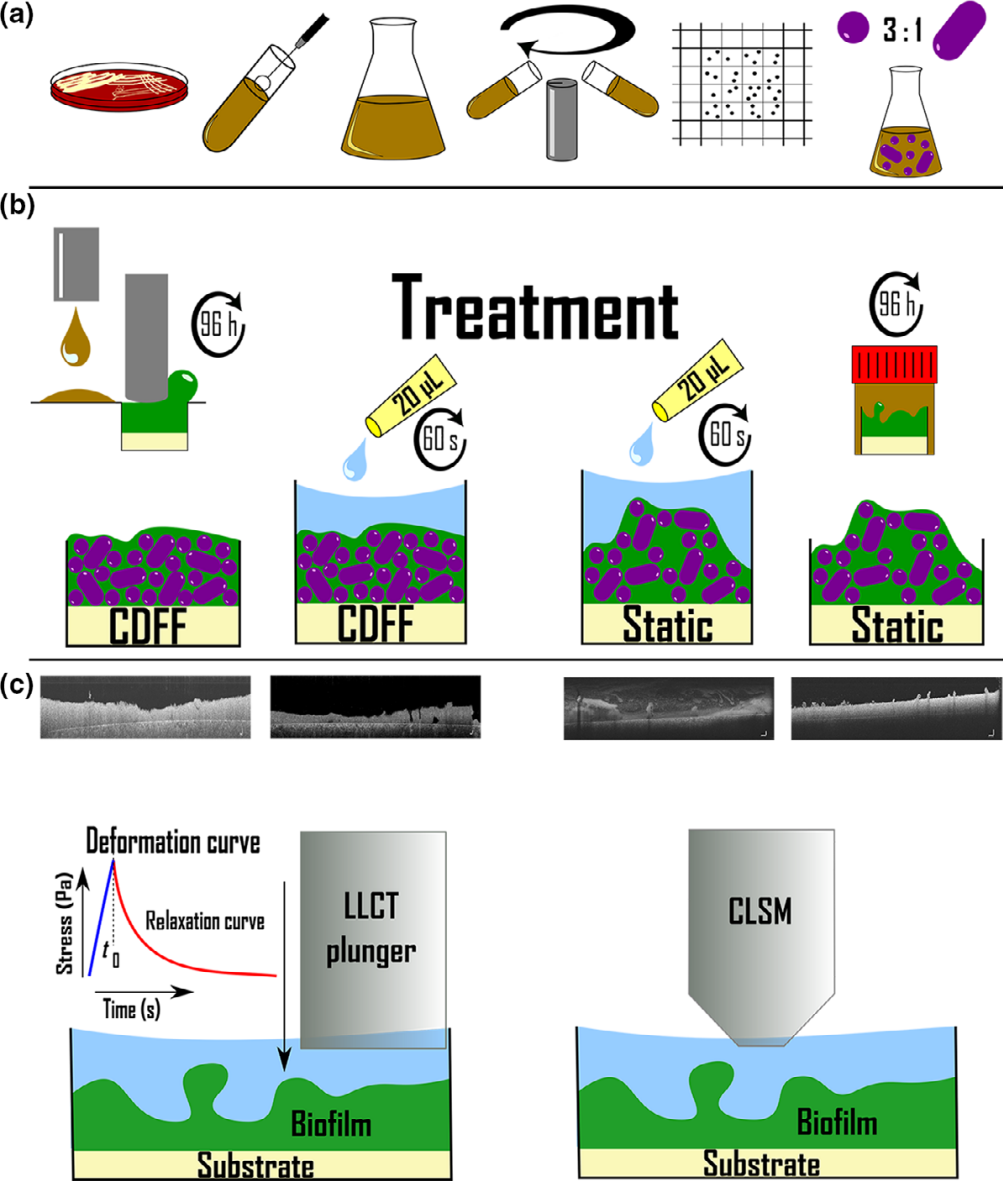
Graphical abstract (concise flowchart) depicting the experimental protocol followed for bacterial culture (a), biofilm growth and treatment (b) and biofilm assessment (c). For details, the reader is referred to Busanello *et al.* 2019 and Petridis *et al.* 2019 (see references, open access articles).

The experimental setup was based on previously described and validated protocols (Busanello *et al.* 2019, Petridis *et al.* 2019) and is briefly presented in a graphical abstract (Fig. 1). Bacterial suspensions of *Streptococcus oralis* J22 (*S. oralis*) and *Actinomyces naeslundii* T14V‐J1 (*A. naeslundii*) were initially cultured in modified brain heart infusion broth (BHI) (37.0 g L^−1^ BHI, 1.0 g L^−1^ yeast extract, 0.02 g L^−1^ NaOH, 0.001 g L^−1^ vitamin K1, 5 mg L^−1^ L‐cysteine‐HCl and pH 7.3) (BHI, Oxoid Ltd., Basingstoke, UK). Next, the bacterial species were co‐cultured at concentrations of 6 × 10^8^ cells mL^−1^ for *S. oralis* and 2 × 10^8^ cells mL^−1^ for *A. naeslundii* for four days on saliva‐coated hydroxyapatite (HA) discs. This led to the formation of defined dual‐species biofilms in terms of thickness and structure (details on bacterial culturing are presented in Busanello *et al.* 2019). Two different dual‐species biofilm types were developed as follows: A four‐day biofilm grown in a constant depth film fermenter (4CDFFB) and a four‐day static biofilm (4SB) grown in confined spaces and under static culturing conditions (details on biofilm growth are presented in Busanello *et al.* 2019). Before any treatment was applied, cross‐sectional scans of the biofilms were acquired with an optical coherence tomography (OCT) scanner (Thorlabs, Newton, NJ, USA) (pre‐treatment scans). During OCT imaging, the biofilms were kept in a volumetric jar with a 20 mL adhesion buffer. The field of view (FOV) was set at 4.5 mm and the refraction index at 1.33, and images were processed with the ThorImage OCT software (Thorlabs). Subsequently, biofilms were transferred to an empty volumetric jar and treated with sterile buffer (0.147 g L^−1^ CaCl_2_, 0.174 g L^−1^ K_2_HPO_4_, 0.136 g L^−1^ KH_2_PO_4_, 3.728 g L^−1^ KCl dissolved in sterile demineralized water, pH 6.8) (control group), 2‐, 5‐, and 10% NaOCl (reagent grade, available chlorine 10–15%, Sigma‐Aldrich, St. Louis, MO, USA). Before every experiment, a thiosulfate titration method was used to determine NaOCl concentration, and accordingly dilution with sterile demineralized water ensued. The treatment consisted of applying 20 μL solution statically (no flow) over the biofilms, followed by a 60 s interval, during which the biofilm samples were left undisturbed. Next, 20 μL sodium thiosulfate (Na_2_S_2_O_3,_ Sigma‐Aldrich) was applied for NaOCl neutralization, and the samples were transferred in a volumetric jar with 20 mL adhesion buffer. The treated biofilms were scanned again with the OCT scanner under the same settings (post‐treatment scans). Quantification of changes on the biofilms was carried out by evaluating the scanned pre‐ and post‐treatment biofilm cross‐sections with an open‐source image analysis software (Fiji, https://imagej.net/Fiji). The distance in every column of pixels between the substrate and top of the biofilm (4,500 rows of pixels) was calculated and compared for the pre‐ and post‐treatment images. To improve the accuracy of the data, different grayscale thresholds in each image were selected (Otsu 1979; Liao *et al.* 2001), resulting in the identification of distinct biofilm layers (Busanello *et al.* 2019). Based on previously validated protocols, the different biofilm layers identified with the OCT were allocated to the terms *disrupted layer* (lower grayscale pixel intensity) and *coherent layer* (higher grayscale pixel intensity) (Busanello *et al.* 2019, Petridis *et al.* 2019). Per cent biofilm dissolution and per cent biofilm disruption were chosen as outcome measures.

For per cent biofilm dissolution, the change of the coherent layer after treatment was calculated using Equation 1.

(1) $$\left[\frac{\text{pre-treatment}\;\text{coherent}\;\text{layer}\;\text{height}\;-\;\text{post-treatment}\;\text{coherent}\;\text{layer}\;\text{height}\;}{\text{pretretment}\;\text{coherent}\;\text{layer}\;\text{height}}\right]\times 100$$

Positive and negative values were related to decrease and increase of the coherent layer height post‐treatment, respectively. Biofilm dissolution was consistent with decrease in the height of the coherent biofilm layer.

For per cent biofilm disruption, the change of the disrupted layer after treatment was calculated using Equation 2.

(2) $$\left[\frac{\text{post-treatment}\;\text{disrupted}\;\text{layer}\;\text{height}\;-\;\text{pre-treatment}\;\text{disrupted}\;\text{layer}\;\text{height}\;}{\text{pretretment}\;\text{disrupted}\;\text{layer}\;\text{height}}\right]\times 100$$

Positive and negative values were related to increase and decrease of the disrupted layer height post‐treatment, respectively. Biofilm disruptionas consistent with increase in the height of the disrupted biofilm layer.

In order to study changes in the biofilm architecture, biofilms treated with NaOCl were subjected to analysis of their viscoelastic properties with the aid of low load compression testing (LLCT). Confocal laser scanning microscopy (CLSM) analysis of stained biofilm components, such as live/dead bacteria and extracellular polysaccharides (EPS), was also employed (details on methodological protocols, data acquisition and analysis are presented in Busanello *et al.* 2019, Petridis *et al.* 2019).

For the visualization of the action of NaOCl, top‐view OCT images of treated biofilms were analysed. Following the 60 s NaOCl application, top‐view OCT snapshots of the whole biofilm sample were captured. Bubble formation was chosen as the outcome measure, quantified by manually counting on the top‐view images the number of the bubbles generated.

Furthermore, in order to gain more insight on the dynamics of the NaOCl bubble formation process over time, biofilm behaviour was registered in real‐time by means of OCT during the subtle continuous flow of the several NaOCl concentrations over four‐day CDFF biofilms (FOV: 4.5 mm, refraction index: 1.33, frame rate: 0.4 image/s). Four‐day static biofilms had an extremely rapid and increased dissolution under the test conditions applied and were therefore excluded from this type of experiment. CDFF biofilm‐carrying HA discs were inserted in a parallel plate flow chamber with the help of a custom‐made silicone mould; this ensured that the biofilm was always placed at the same level with regard to its vertical protrusion in the chamber, and parallel to the chamber surface and irrigant flow. Next, buffer (control), 2‐, 5‐ and 10% NaOCl were introduced at a low flow rate (3.33 mL min^−1^), and real‐time recording with the OCT scanner was performed for 60 s. Three biofilm samples per treatment group were used during three independent experiments.

For each biofilm evaluation technique applied (OCT, LLCT and CLSM), 20 samples from each biofilm type were divided into four groups according to the treatment provided (control, 2‐, 5‐ and 10% NaOCl). Statistical analysis was performed using SPSS software (version 23.0, IBM Corp., Armonk, NY, USA). Normality of data was assessed through the Shapiro‐Wilk test. One‐way analysis of variance (ANOVA) (or Welch’s ANOVA) was carried out to detect the presence of significant differences among the various treatments employed for each biofilm type. Tukey’s HSD (or Games‐Howell) *post‐hoc* multiple comparison tests were subsequently performed to identify significant differences between the several chemical treatments. Linear regression analysis was performed to test the correlation between bubble formation (number of bubbles and dependent variable) and NaOCl concentration (predictor variable). Data are presented as mean and standard deviation (SD). The level of statistical significance was set at *a* ≤ 0.05.

## Results

### Anti‐biofilm efficacy of NaOCl concentrations assessed with optical coherence tomography

#### Four‐day CDFF biofilms (4CDFFB)

**Figure 2:**
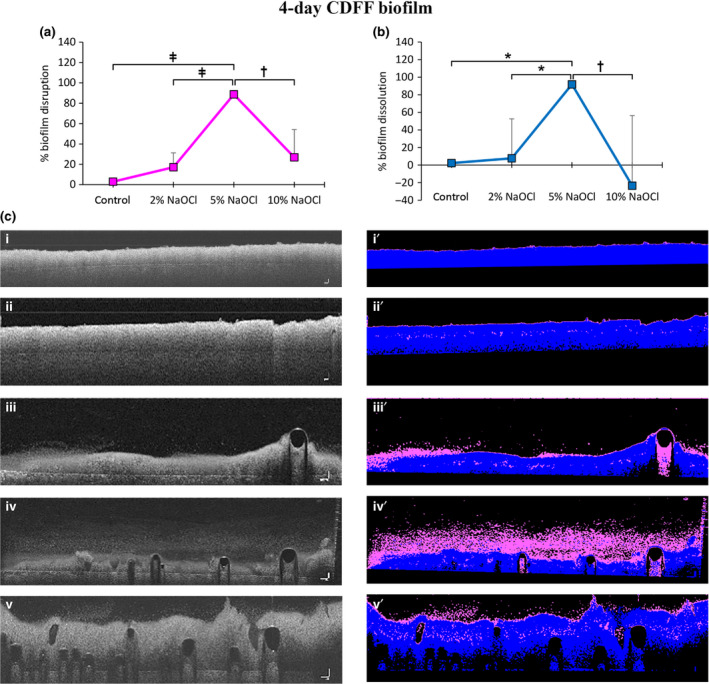
Biofilm disruption (a) and dissolution (b) for four‐day CDFF biofilms after 60 s treatment with buffer (control), 2‐, 5‐ and 10% NaOCl. Values are presented as means with error bars representing standard deviation. Statistical significance is represented by * for *P* ≤ 0.05, † for *P* ≤ 0.01 and ‡ for *P* ≤ 0.001. Representative cross‐sectional greyscale OCT scans of four‐day CDFF biofilms and the respective processed images based on the grayscale level thresholding applied; pseudocolors added to highlight the remaining coherent biofilm layer (blue) and resultant disrupted biofilm layer (purple) after the 60 s treatment with the several solutions (c); greyscale pre‐treatment scan (i) and respective processed image (i´); greyscale buffer post‐treatment scan (ii) and respective processed image (ii´); greyscale 2% NaOCl post‐treatment scan (iii) and respective processed image (iii´); greyscale 5% NaOCl post‐treatment scan (iv) and respective processed image (iv´); greyscale 10% NaOCl post‐treatment scan (v) and respective processed image (v´). Scale bars represent 100 μm.

Treatment with 5% NaOCl significantly increased biofilm disruption compared to the control (*P* < 0.001), 2% NaOCl (*P* < 0.001) and 10% NaOCl (*P* < 0.001) (Fig. 2a). It also increased biofilm dissolution compared to the control (*P* = 0.032), 2% NaOCl (*P* = 0.046) and 10% NaOCl (*P* = 0.005).

The mean per cent biofilm dissolved in the 10% NaOCl treatment group yielded a negative value, indicating an increase in the height of the coherent biofilm layer (Fig. 2b).

Representative OCT scans of biofilms pre‐ and post‐treatment are presented in Fig. 2c.

#### Four‐day static biofilms (4SB)

**Figure 3:**
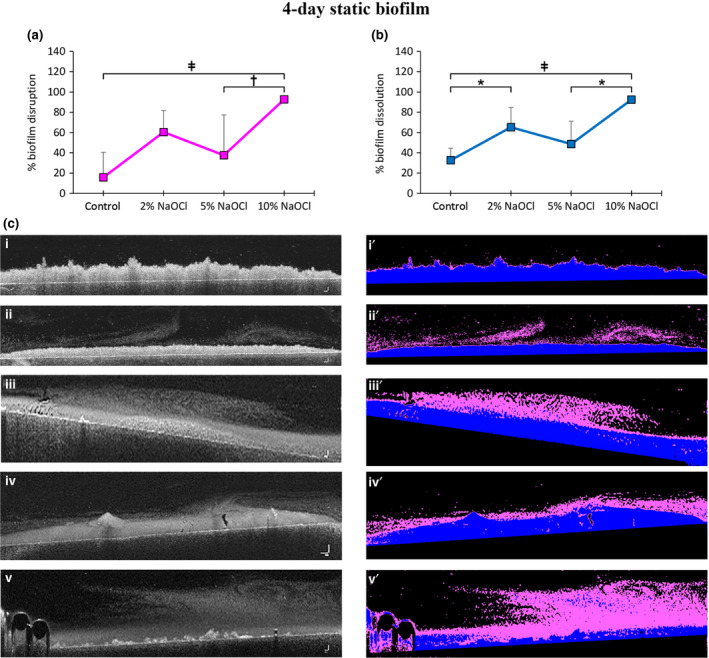
Biofilm disruption (a) and dissolution (b) for four‐day static biofilms after 60 s treatment with buffer (control), 2‐, 5‐ and 10% NaOCl. Values are presented as means and error bars representing standard deviation. Statistical significance is represented by * for *P* ≤ 0.05, † for *P* ≤ 0.01 and ‡ for *P* ≤ 0.001. Representative cross‐sectional greyscale OCT scans of four‐day static biofilms and the respective processed images based on the grayscale level thresholding applied; pseudocolors added highlight the remaining coherent biofilm layer (blue) and resultant disrupted biofilm layer (purple) after 60 s treatment with the several solutions (c); greyscale pre‐treatment scan (i) and respective processed image (i´); greyscale buffer post‐treatment scan (ii) and respective processed image (ii´); greyscale 2% NaOCl post‐treatment scan (iii) and respective processed image (iii´); greyscale 5% NaOCl post‐treatment scan (iv) and respective processed image (iv´); greyscale 10% NaOCl post‐treatment scan (v) and respective processed image (v´). Scale bars represent 100 μm.

Treatment with 10% NaOCl significantly increased biofilm disruption compared to the control (*P* = 0.001) and 5% NaOCl (*P* = 0.018), while disrupting considerably more biofilm compared to 2% NaOCl (Fig. 3a). It also significantly increased biofilm dissolution compared to the control (*P* < 0.001) and 5% NaOCl (*P* = 0.003), while dissolving considerably more biofilm compared to 2% NaOCl.

Treatment with 2% NaOCl significantly increased biofilm dissolution compared to the control (*P* = 0.026) (Fig. 3b).

Representative OCT scans of four‐day static biofilms pre‐ and post‐treatment are presented in Fig. 3c.

### Effect of NaOCl concentration on biofilm architecture: low load compression testing

#### Four‐day CDFF biofilms (4CDFFB)

**Figure 4:**
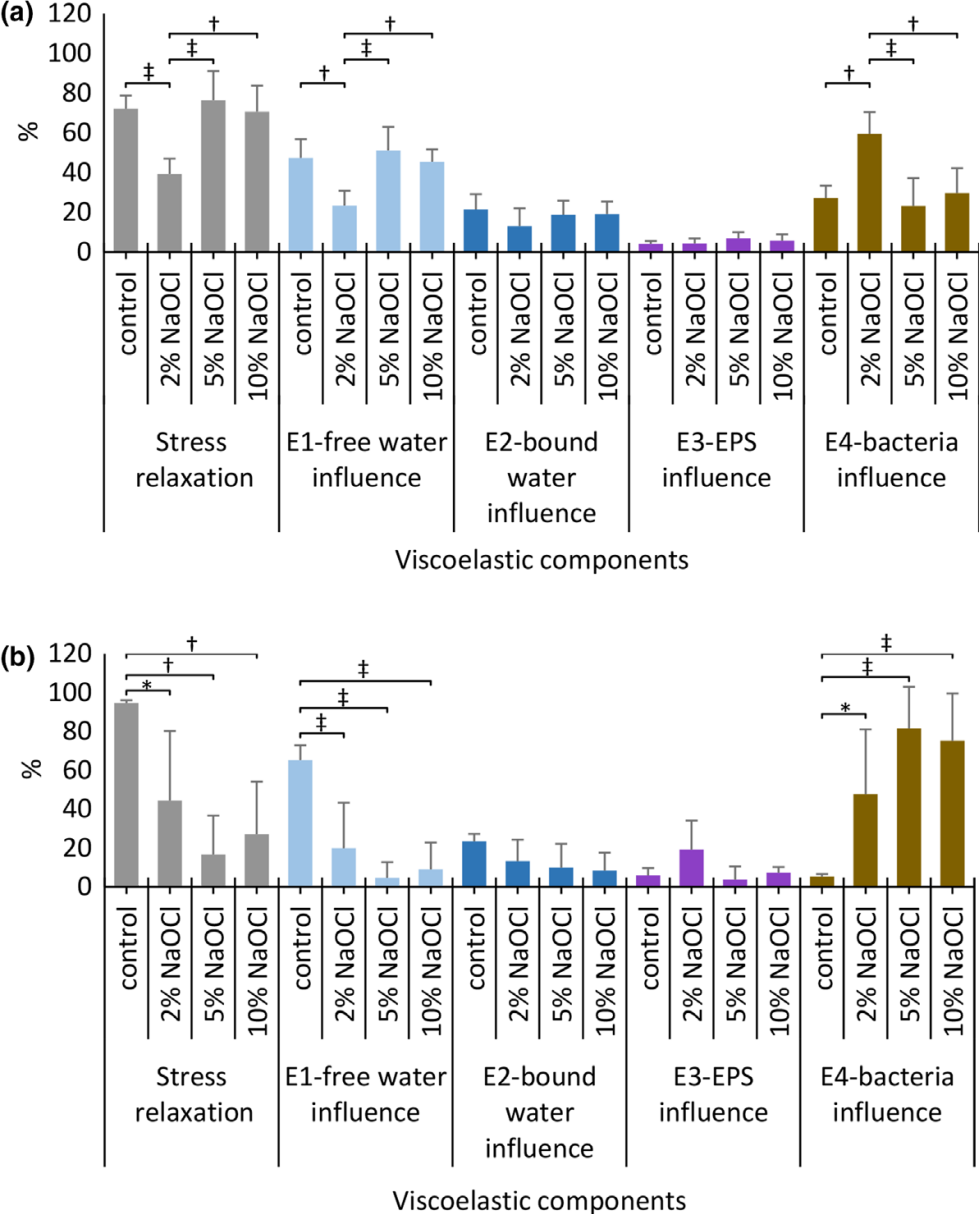
Low load compression testing‐derived biofilm viscoelasticity profile after 60 s treatment with buffer (control), 2‐, 5‐ and 10% NaOCl for four‐day CDFF (a) and four‐day static (b) biofilms. The y‐axis represents per cent stress relaxation and influence of the 4 Maxwell element (E1, E2, E3 and E4). Each Maxwell element was associated with a unique component of the biofilm structure (free water, bound water, EPS and bacteria). Values are presented as means with error bars representing standard deviation. Statistical significance is represented by * for *P* ≤ 0.05, † for *P* ≤ 0.01 and ‡ for *P* ≤ 0.001.

Treatment with 2% NaOCl caused a significant decrease in the stress relaxation compared to the control (*P* = 0.001), 5% NaOCl (*P* < 0.001) and 10% NaOCl (*P* = 0.002) (Fig. 4a). The mathematical fitting of the generated stress relaxation curves using a generalized Maxwell model revealed:

- significant decrease of the relative importance of the E_1_ Maxwell element (representing the free water biofilm component, see Busanello *et al.* 2019) in the 2% NaOCl‐treated remaining biofilms compared to the control (*P* = 0.003), 5% NaOCl (*P* = 0.001) and 10% NaOCl (*P* = 0.007) (Fig. 4a) and
- significant increase of the relative importance of the E_4_ Maxwell element (representing the bacterial cell biofilm component, see Busanello *et al.* 2019) in the 2% NaOCl‐treated remaining biofilms compared to the control (*P* = 0.002), 5% NaOCl (*P* = 0.001) and 10% NaOCl (*P* = 0.004) (Fig. 4a).

The relative importance of the E_2_ and E_3_ Maxwell elements in the four‐day CDFF biofilms (representing the bound water and EPS biofilm respectively) were not significantly affected, irrespective of the treatment applied (Fig. 4a).

#### Four‐day static biofilms (4SB)

Treatment with NaOCl, irrespective of the concentration used, caused a significant decrease in the stress relaxation, compared to the control (*P* = 0.024 for 2% NaOCl, *P* = 0.001 for 5% NaOCl and *P* = 0.003 for 10% NaOCl) (Fig. 4b). No significant differences among NaOCl concentrations were detected. The mathematical fitting of the generated stress relaxation curves using a generalized Maxwell model revealed:

- a significant decrease of the relative importance of the E_1_ Maxwell element (representing the free water biofilm component, see Busanello *et al.* 2019) in all NaOCl‐treated remaining biofilms, irrespective of the concentration used, compared to the control (*P* = 0.001 for 2% NaOCl and *P* < 0.001 for 5% and 10% NaOCl), as well as no significant differences among the several NaOCl concentrations (Fig. 4b) and
- a significant increase of the relative importance of the E_4_ Maxwell element (representing the bacterial cell biofilm component, see Busanello *et al.* 2019) in all NaOCl‐treated remaining biofilms, irrespective of the concentration used, compared to the control (*P* = 0.049 for 2% NaOCl and *P* < 0.001 for 5% and *P* = 0.001 for 10% NaOCl), as well as no significant differences among the several NaOCl concentrations (Fig. 4b).

The relative importance of the E_2_ and E_3_ Maxwell elements in the 4‐day static biofilms (representing the bound water and EPS components of the biofilms, respectively) were not significantly affected, irrespective of the treatment applied (Fig. 4b).

### Effect of NaOCl concentration on biofilm architecture: confocal laser scanning microscopy

#### Four‐day CDFF biofilms (4CDFFB)

**Figure 5:**
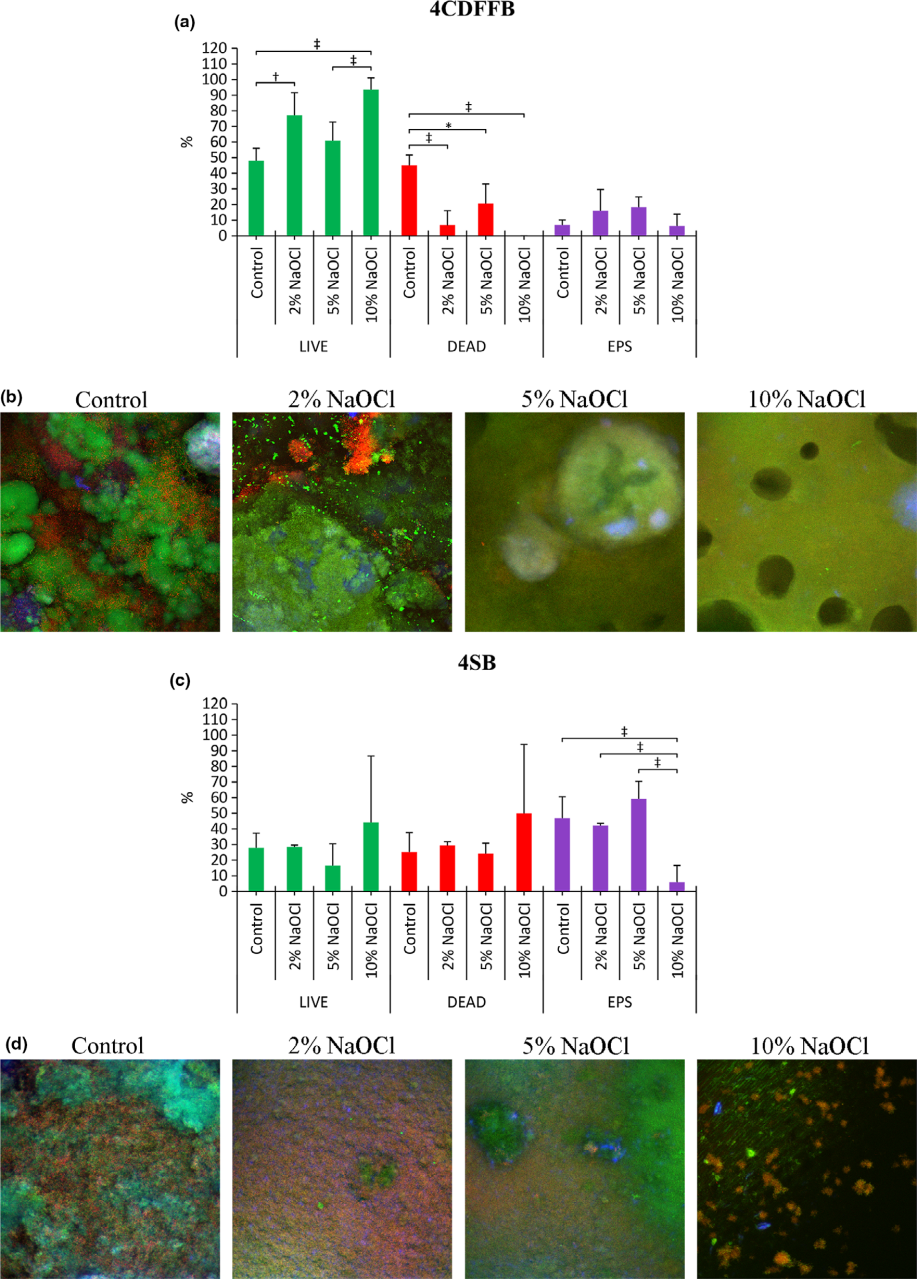
Confocal laser scanning microscopy (CLSM) biofilm architectural profile after 60 s treatment with buffer (control), 2‐, 5‐ and 10% NaOCl for four‐day CDFF biofilms (4CDFFB) (a, b) and for four‐day static biofilms (4SB) (c, d). In the bar graphs, the y‐axis represents per cent stained live bacteria, dead bacteria and extracellular polysaccharides (EPS). Values are presented as means with error bars representing standard deviation. Statistical significance is represented by * for *P* ≤ 0.05, † for *P* ≤ 0.01 and ‡ for *P* ≤ 0.001.

Treatment of four‐day CDFF biofilms with NaOCl had a significant impact on the bacterial cell biofilm component, without significantly affecting the EPS biofilm component. Per cent ‘LIVE’ bacteria was significantly higher after treatment with 10% NaOCl, compared to 5% NaOCl (*P* = 0.001) and the control (*P* < 0.001). Also, treatment with 2% NaOCl resulted in a significantly higher per cent ‘LIVE’ bacteria compared to the control (*P* = 0.003) (Fig. 5a). Per cent ‘DEAD’ bacteria was significantly reduced compared to the control, irrespective of the NaOCl concentration used (*P* = 0.001 for 2% NaOCl, *P* = 0.03 for 5% NaOCl and *P* < 0.001 for 10% NaOCl), while no significant differences were detected among the NaOCl groups (Fig. 5a).

#### Four‐day static biofilms (4SB)

Treatment with NaOCl showed insignificant changes regarding their bacterial cell component. Significant changes were detected in the EPS biofilm component, where treatment with 10% NaOCl resulted in a significant reduction of EPS compared to the control (*P* < 0.001), 2% NaOCl (*P* < 0.001) and 5% NaOCl (*P* < 0.001) (Fig. 5b).

### Evaluation of NaOCl‐induced bubble formation: snapshot rendering after static application

**Figure 6:**
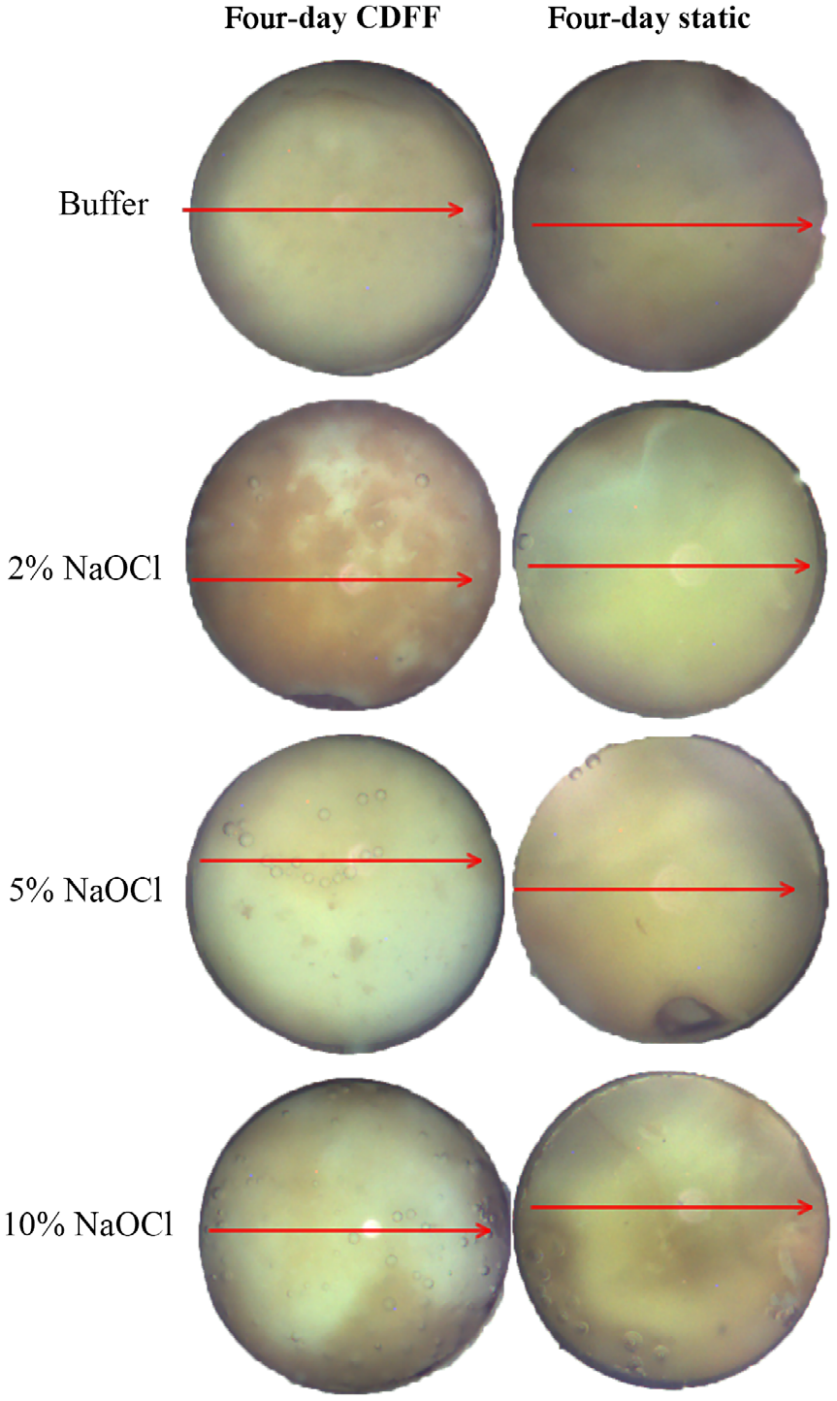
Top‐view biofilm snapshots after 60 s treatment with buffer (control), 2‐, 5‐, and 10% NaOCl for four‐day CDFF and static biofilms. Images were acquired with the optical coherence tomography scanner prior to acquisition of the cross‐sectional grayscale biofilm images (red arrows indicate the scanning direction). Bubble formation is evident with increasing NaOCl concentration in the four‐day CDFF biofilms and mainly in the 10% NaOCl treatment group for the four‐day static biofilms.

Descriptive statistics are presented in Table 1. Analysis of the number of bubbles visible in the top‐view OCT images of the four‐day CDFF biofilms treated revealed that 10% NaOCl generated significantly more bubbles compared to control (*P* < 0.001), 2% NaOCl (*P* < 0.001) and 5% (*P* < 0.001) (Fig. 6). The linear regression analysis revealed significant correlation between the NaOCl concentration and the amount of bubbles formed, described in the following function:

**Table 1:** Bubble count mean values (standard deviation within brackets) present in the top‐view OCT images of the four‐day CDFF biofilms treated with NaOCl

Treatment	Outcome measures	
	Four‐day CDFF biofilm Bubble count (SD)	Four‐day static biofilm Bubble count (SD)
Control (buffer)	0.0 (0.0)	0.0 (0.0)
2% NaOCl	2.7 (3.6)	1.2 (1.1)
5% NaOCl	13.6 (14.2)	1.1 (1.1)
10% NaOCl	44.6 (21.2)	10.8 (10.1)

*N*_bubbles_ = 4.6 × *C*_NaOCl_ ‐ 3.7, (*R* ^2^ = 0.812, *F* = 75.3, *P* < 0.001), where *N*_bubbles_ = amount of bubbles formed and *C*_NaOCl_ = NaOCl concentration.

For the four‐day static biofilms, treatment with 10% NaOCl generated significantly more bubbles compared to control (*P* = 0.020), 2% NaOCl (*P* = 0.043) and 5% (*P* = 0.022). The linear regression analysis did not reveal any linear correlation between NaOCl concentration and the amount of bubbles formed.

### Evaluation of NaOCl‐induced bubble formation: Real‐time rendering during irrigant flow

Bubble formation was clearly observed with the real‐time OCT video recording of CDFF biofilm samples exposed to subtle irrigant flow in the parallel plate flow chamber, creating the impression that higher NaOCl concentrations generate more, larger and faster bubble formation (Videos S1, S2, S3, S4).

## Discussion

To study the chemical anti‐biofilm efficacy and action of several sodium hypochlorite (NaOCl) concentrations, micro‐volume NaOCl solutions were applied statically over two structurally defined biofilms for a finite time interval (60 s). In the present study, NaOCl action was dependent solely on diffusion (and not convection). In that sense, factors that could alter the process of NaOCl diffusion into the bulk biofilm besides concentration, such as the biofilm structure, NaOCl reactivity with the biofilm matrix and time allowed for NaOCl diffusion (60 s) should be also taken into consideration.

Biofilm disruption should be viewed as the aftermath of the immediate reaction between the prevailing oxidizing hypochlorite (OCl^‐^) and the extracellular polymeric substances of the biofilm matrix, such as proteins and polysaccharides (Baker 1947, Tawakoli *et al.* 2015) and forerunner of biofilm dissolution. This rapidly occurring chemical reaction leads to generation of bubbles, whose gas content is mainly composed of carbon dioxide and chloroform compounds (Mohmmed 2017). These chloroform compounds are possibly reaction products of the oxidation of the polymeric content of the biofilm matrix and/ or the peptidoglycans (cell wall component of the Gram‐positive bacteria used in this study) by hypochlorous acid (HOCl^‐^) of NaOCl (Hawkins *et al.* 2003). Bubble formation seems to cause a collapse of the biofilm structure which depending on the unique biofilm architecture, facilitates dissolution and/ or mechanical removal at a different degree.

In the structurally compacted four‐day CDFF biofilms 5% NaOCl caused significant biofilm disruption and dissolution compared to 2% and 10% solutions. Two per cent NaOCl barely affected this biofilm type, while surprisingly, 10% NaOCl resulted in significantly impaired biofilm disruption and dissolution (negative mean values of per cent dissolution). By taking a closer look at the behaviour of the CDFF biofilms using OCT scans and snapshot images, an increase in the biofilm height (Fig. 2d) and an increased amount of bubbles could be seen in 10% NaOCl treatment CDFF biofilm group (Fig. 6a). Apparently, this typical chemical reaction induced by the 10% NaOCl, in combination with a compact biofilm structure, resulted in the formation of bubbles capable of lifting up the entire biofilm from its underlying substrate (Video S4). This suggests that 10% NaOCl is capable of penetrating deeper in the bulk biofilm (increased diffusion), thus bringing about this gas‐associated bubble formation at the biofilm‐substrate interface. The upward pushing force accounted for the increased biofilm height, and thereby negative mean values noted after application of 10% NaOCl (Fig. 2b).

With regard to the structurally less compacted four‐day static biofilms, the expected increased anti‐biofilm efficacy with increasing NaOCl concentration was only partially confirmed. In contrast to the dense CDFF biofilms, the increased anti‐biofilm capacity of the 10% NaOCl was clearly noticeable within the 60 s application interval, leading almost to complete biofilm disruption and dissolution. Moreover, the associated bubble count was significantly higher compared to the lower concentrations, indicating a stronger chemical effect resulting in gas‐associated bubble formation. Interestingly, 2‐ and 5% NaOCl concentrations did not demonstrate a significant difference in their disruptive and dissolving capacity, as opposed to their significant effect on the CDFF biofilms. Additionally, induction of bubble formation was barely noticeable and equally low in these groups.

The findings presented above suggest that biofilm structure drives the chemical interplay between the oxidizing reagent and the underlying biofilm and eventually the anti‐biofilm efficacy of NaOCl. Specifically, the four‐day static biofilms had a loose architecture compared to the four‐day CDFF biofilms as a result of the decreased bacterial density, increased EPS presence and significantly increased amount of ‘free’ water (Busanello *et al.* 2019). When this open biofilm architecture is exposed to concentrated NaOCl solutions, penetration of the biocide is favored, and the chemical interaction with deeper biofilm layers is facilitated (Stewart 2003). This explains the enhanced anti‐biofilm efficacy and bubble formation associated with 10% NaOCl on the four‐day static biofilms. However, the lack of any noticeable difference between the 2‐ and 5% NaOCl indicates a concentration range within which the anti‐biofilm efficacy plateaus. It could be argued that due to the increased amount of EPS present, an increased reactivity, and hence, NaOCl consumption occurs. This creates a diffusion barrier that intermediate concentrations cannot overcome, thus accounting for the comparable lower anti‐biofilm efficacy of the 2‐ and 5% NaOCl.

Quantification of bubbles formed was performed on top‐view OCT images captures after 60 s of NaOCl application. Therefore, bubble count was an end‐point measurement performed on OCT snapshots. It needs to be stressed out that the generation of bubbles is a dynamic process dictated by the reactivity between the oxidative reagent and the underlying biofilm. This starts immediately after NaOCl application and progresses over time. Also, it is reasonable that when a considerable amount of biofilm has been already dissolved due to the action of NaOCl, the amount of bubbles counted on a later time point will be less. This leads eventually to a lower bubble count in the four‐day static compared to the four‐day CDFF biofilms, and hence to an underestimation of the chemical process that actually takes place. To conclude, the end‐point measurement of bubble count provides valuable information regarding the chemical action of NaOCl on a biofilm substrate but specific limitations need to be accounted for. Real‐time OCT video analysis or high‐speed microscopy could possibly help resolve limitations associated with time end‐point measurements.

Arguably, the low reactivity of the 2% NaOCl with the poor EPS content of the upper layers of the four‐day CDFF biofilms causes initially superficial changes leading to the formation of small bubbles. As the bubbles leave the biofilm out they cause evaporation of the thin layer of ‘free’ water, while displacing and packing bacteria to adjacent regions (Jang *et al.* 2017). This is supported by the significant decrease of the influence free water component (E_1_) and increase of the influence of the bacterial component (E_4_) noted for the 2% NaOCl‐treated CDFF biofilms. By increasing NaOCl concentration to 5%, penetration is increased and a chemical reaction with the deeper situated EPS occurs. This results in the formation of more bubbles in deeper layers (Video S3), which in turn increases the chances of permanent attachment of pieces of biofilm to the moving bubble (Walls *et al.* 2014). Thus, the likelihood of biofilm cohesion failure is increased and along with that the bubble‐driven biofilm disruption and subsequent removal from the inner layers become more effective. The increased biofilm dissolution noted corroborates this hypothesis.

Application of 10% NaOCl leads to deeper penetration of the reagent and consequently to significantly more bubble formation in the innermost layers of the four‐day CDFF biofilms. A fast‐occurring chemical reaction resulting in the formation of a significant amount of bubbles of larger dimensions (as compared to the bubbles generated from the action of the lower NaOCl concentrations) is illustrated (Video S4). These large bubbles seem to emerge mostly from the interface between the biofilm and the HA discs. During their upward course, they detach and carry with large pieces of biofilm from the underlying substrate, hence causing biofilm adhesion failure. This is also illustrated in the post‐treatment OCT scans. Nonetheless, as elaborated previously, the quantitative data analysis creates the impression that 10% NaOCl is inefficient in biofilm removal. Notably, when a subtle flow rate is also applied, the remarkable anti‐biofilm efficacy of 10% NaOCl is clearly evident (Video S4).

The CLSM findings for the four‐day CDFF biofilm support the argument above and are consistent with the structural features of this biofilm type. Significant changes were detected only in the bacterial cell component of the biofilm (predominant biofilm component), while EPS remained mostly unaffected (less predominant biofilm component). Indeed, in the 10% NaOCl‐treated CDFF biofilms, per cent LIVE and DEAD bacteria were the highest and lowest respectively, compared to the other groups. This indicates that a chemical effect other than bacterial killing took place and is also in accordance with the biofilm detachment observed.

In the four‐day static biofilms, the richer EPS content increases the reactivity sites with NaOCl, thereby limiting its penetration. Interestingly, for the 2‐ and 5% NaOCl, reactivity with the EPS seemed to be similarly low, as indicated by the lack of significant difference in biofilm disruption (OCT findings) and presence of EPS (CLSM findings) in the treated biofilms. The significant amount of water present in this biofilm type (Busanello *et al.* 2019) could account for this finding, that is, despite the higher concentration of the 5% NaOCl, the presence of water brings about a dilution effect, thereby decreasing its anti‐biofilm efficacy. In contrast, application of 10% NaOCl seemed to affect profoundly the four‐day static biofilms. This was demonstrated by the increased biofilm disruption and dissolution induced by 10% NaOCl, as well as the significantly lower presence of EPS noted in the 10% NaOCl‐treated biofilms. Moreover, the increased bubble count recorded in this group signifies an intensified chemical interaction between the abundant OCl^‐^ and the EPS.

Furthermore, the open biofilm structure of four‐day static biofilms facilitates the movement of the bubbles generated during application of NaOCl. As a result, evaporation of free water and bacterial packing during treatment, as described previously, possibly occur. This is reflected to the viscoelastic properties of the biofilms, as evidenced by the decreased E_1_ and increased E_4_ Maxwell elements, respectively. However, contrary to the four‐CDFF biofilms where these phenomena were associated only with the 2% NaOCl, the four‐day static biofilms were similarly affected, irrespective of the NaOCl concentration applied. This highlights once again the influence of the architecture of the initial biofilm on the action of NaOCl exerted.

Taking into account the diffusion limitations imposed by the different architecture of the biofilms used and the overall results of this study, the following theory is presented in an attempt to explain the behaviour of both biofilm types to the several NaOCl concentrations applied:

Sodium hypochlorite exhibits limited penetration due to the immediate NaOCl consumption that occurs from the reaction of the reagent with the organic biofilm substrate (Stewart *et al.* 2001, Stewart 2003). This effect is pronounced at low concentrations of NaOCl, the biofilm thickness increases and, arguably, as the biofilm structure make‐up poses inherent obstacles that hinder diffusion. Four‐day CDFF biofilms present a bacterial dense structure which is low in water and EPS content, whereas four‐day static biofilms are highly hydrated, with a significantly less tight bacterial backbone and more EPS (Busanello *et al.* 2019). The dense bacterial aggregation and the low water content impede deeper transport of solutes, thereby limiting NaOCl penetration. At the same time, the low water content decreases the dilution effect on NaOCl concentration, while the low EPS content offers less sites for chemical interactions. These should result in deeper NaOCl penetration in the bulk biofilm as NaOCl concentration increases. By comparison, while a less bacterial‐tight biofilm architecture facilitates solute transportation, the abundant presence of water and EPS pose diffusivity barriers due to the dilution effect and reactivity of NaOCl with the EPS (Stewart 2003). These barriers create a ‘concentration plateau’ (2‐ to 5% NaOCl), only above which the anti‐biofilm efficacy of NaOCl is clearly evident (10% NaOCl). To summarize, apart from factors related to NaOCl application that are amenable to fine‐tuning by the operator (e.g. concentration, time, volume, temperature and agitation), the biofilm architecture emerges as an important regulator of NaOCl penetration, and hence of its anti‐biofilm efficacy.

Bubble formation is anticipated when a strong oxidizing reagent (NaOCl) comes in contact with an organic substrate (biofilm). Nevertheless, this is the first study linking bubble growth to biofilm removal and NaOCl concentration. Increasing NaOCl resulted in an increase in the amount of bubbles generated, irrespective of the biofilm type. While the generalized notion supporting that by increasing NaOCl concentration the chemical reactions with the underlying biofilm are potentiated and prolonged holds true, factors that influence bubble coalescence should not be overlooked. It has been demonstrated that the mechanism of bubble coalescence is strongly dependent on the presence of specific electrolytes and their concentration in aqueous solutions (Craig *et al.* 1993). Recent studies using dynamic models of bubble formation (i.e. conditions close to the bubble growth behaviour observed after NaOCl application on biofilms) concluded that the increased presence of ions among fast‐approaching bubbles delays or inhibits the naturally occurring bubble coalescence phenomenon. This occurs as a result of the development of electro‐repulsive forces developed between the thin film separating the bubbles (Yaminsky *et al.* 2010, Katsir & Marmur 2014, Katsir *et al.* 2015).

Applying these findings to the outcome of bubble formation as illustrated in the present study, the following could be argued: an increased concentration of NaOCl makes up an ion‐abundant environment. As the bubbles that are generated from the reaction with the biofilm approach to each other, the rich ionic environment inhibits them from coalescing. As a consequence, the stable bubbles remain for a longer time within the bulk biofilm without merging, thereby contributing to the enhanced biofilm adhesion and/ or cohesion failure observed in this study when the concentrated NaOCl solution was applied.

## Conclusions

In the present study, two types of dual‐species biofilms representing different bacterial communities in terms of water content, EPS presence, bacterial density and viscoelastic properties were challenged with several concentrations of NaOCl and the diffusion‐dependent effects of the biocide were investigated. In general, by increasing NaOCl concentration, its anti‐biofilm efficacy was enhanced, with distinct biofilm removal patterns standing out within each biofilm type. The findings suggested that the architecture of the biofilm to treat should be acknowledged as an additional factor in the equilibrium that determines the penetration capacity of NaOCl, and as a consequence its potential to affect and remove biofilms. Optical coherence tomography appeared to be a suitable technique to analyze end‐point outcomes of the biofilm fate after treatment with NaOCl. It also provided illustrative information on the bubble‐forming action of NaOCl on biofilms and how this action is linked to biofilm removal. Certain imaging limitations were highlighted and solutions to circumvent them were proposed as a means to explore in‐depth the working mechanisms of NaOCl (e.g. real‐time OCT imaging and high‐speed microscopy). The study of the viscoelastic properties combined with confocal laser scanning microscopy analysis of the remaining biofilms provided supplemental information in support of certain hypotheses about the interpretation of the findings presented. Lastly, a theory about the effect of the concentration of NaOCl on the stability of the bubbles generated was proposed based on bubble coalescence models.

## Conflict of interest

Dr. Busanello and Prof. Dr. So were financially supported by a CNPq scholarship, and a part of the study was financed by a Research Grant of the European Society of Endodontology (ESE). All other authors state explicitly that there are no conflicts of interest in connection with this article.

## Supporting information


**Video S1.** Real‐time rendering (60 seconds, playback is speed‐up 2x) showing a four‐day mature *S. oralis* J22/ *A. naeslundii* T14V‐J1 biofilm grown in a constant depth film fermenter during introduction of buffer at a flow rate 3.33 mL min^-1^ in a parallel plate flow chamber (flow direction is from right to left). No biofilm removal or bubble formation is evident.

**Video S2.** Real‐time rendering (60 seconds, playback is speed‐up 2x) showing a four‐day mature *S. oralis* J22/ *A. naeslundii* T14V‐J1 biofilm grown in a constant depth film fermenter during introduction of 2% NaOCl at a flow rate 3.33 mL min^-1^ in a parallel plate flow chamber (flow direction is from right to left).

**Video S3.** Real‐time rendering (60 seconds, playback is speed‐up 2x) showing a four‐day mature *S. oralis* J22/ *A. naeslundii* T14V‐J1 biofilm grown in a constant depth film fermenter during introduction of 5% NaOCl at a flow rate 3.33 mL min^-1^ in a parallel plate flow chamber (flow direction is from right to left).

**Video S4.** Real‐time rendering (60 seconds, playback is speed‐up 2x) showing a four‐day mature *S. oralis* J22/ *A. naeslundii* T14V‐J1 biofilm grown in a constant depth film fermenter during introduction of 10% NaOCl at a flow rate 3.33 mL min^-1^ in a parallel plate flow chamber (flow direction is from right to left).
